# Feasibility of using risk prompts to prevent falls, dehydration and pulmonary aspiration in nursing homes: a clinical study protocol

**DOI:** 10.1186/s40814-018-0236-1

**Published:** 2018-01-25

**Authors:** Márcia Duarte, Raquel Bouça-Machado, Josefa Domingos, Catarina Godinho, Joaquim J. Ferreira, on behalf of CNS risk prompt display study group, Natália Pona, João Ferreira, Hélder Marques, Tiago Reis, Marta Pires, Ana Monteiro, Eduarda Cabral, Élia Decoroso, José Resende, Margarida Duarte, Matilde Mesquita, Ricardo Gonçalinho, Ricardo Neves, Silvana Costa, Valentina Orghian, Daniela Guerreiro, Alexandra Saúde, Diana Peralta, Francisco Queimado, João Belo, Mariana Leitão, Pedro Nunes, Verónica Caniça, Rita Cardoso, Ana Fernandes, Joana Carvalho, Diana Miranda, Abigail Cascais, Ana Ferreira, Ana Caetano, Ana Santos, Aúrea Filipe, Aurica Gangan, Carmn Vieira, Cátia Franco, Célia Alves Cláudia Marouco, Cristiane Oliveira, Diogo Costa, Dovilé Galdikaite, Elisabete Gonçalves, Elsa Ribeiro, Fátima Carmo, Franclin Oliveira, Gonçalo Louro, Isabel Constantino, Joana Rosa, Joana Maltezinho, Glória Santos, Fátima Gama, Jacinta Louro, Teresa Penetra, Marie Lim, Marília Paulo, Marina Roque, Michele Miranda, Patrícia Elias, Paula Sousa, Rafaela Cordeiro, Sandra Silva, Sandra Santos, Sara Silva, Tatiana Ramos

**Affiliations:** 1 CNS-Campus Neurológico Sénior, Torres Vedras, Portugal; 2 Instituto de Medicina Molecular, Lisbon, Portugal; 3 Centro de Investigação Interdisciplinar Egas Moniz, Escola Superior de Saúde Egas Moniz, Monte de Caparica, Portugal; 4 Laboratory of Clinical Pharmacology and Therapeutics, Faculty of Medicine, University of Lisbon, Lisbon, Portugal

**Keywords:** Nursing homes, Falls, Dysphasia, Pulmonary aspiration, Choking, Dehydration

## Abstract

**Background:**

Evidence has shown a relationship between dehydration, falls, and pulmonary aspiration among older adults in nursing homes, all of which contribute to loss of independence and quality of life. It is believed that improving communication among healthcare professionals in nursing homes (physicians, nurses, rehabilitation team, psychologist, social workers, dieticians and medical assistants) decreases the number of adverse events in institutionalized patients. This study will evaluate the feasibility of using a set of written signs, designed to caution against the risk of falls, dehydration, and pulmonary aspiration, and will enable the proposal of tailored interventions to manage these events in nursing homes.

**Methods/Design:**

All patients from Campus Neurológico Sénior (CNS) nursing home, at risk of falls and/ordysphagia and/or dehydration will be invited to participate in the study. Patients will undertake a screeningrisk assessment and the corresponding risk prompts will be attributed. Study duration will be a minimum ofthree months per participant, including daily record of falls, dehydration and pulmonary aspiration eventsand monthly interview assessments, conducted by a member of the research team. Data of the events that occur will be compared with historical data extracted retrospectively from medical and nursing charts. This study has been approved by the Ethics Committee of the Medical Academic Center of Lisbon, Faculty of Medicine, University of Lisbon (Ref. 176/15). All participants will give their written informed consent before entering the study.

**Discussion:**

This study is unique in evaluating the feasibility of a communication system in preventing the three major risks in nursing home. Thoughtful selection and display of proper risk prompts in nursing homes could be an essential step along a path toward efficient communication of risks among healthcare teams. We expect that the displays will be easily applicable given their simplicity, low complexity, and minimal physical requirements.

**Trial registration:**

NCT03123601. March 7, 2017. Retrospectively registered.

## Background

Due to demographic ageing and increased life expectancy, an increasing number of elderly spend the end of their life in an institutional setting [1].

Nursing homes provide 24-h nursing care to residents with heterogeneous diagnoses, different degrees of functional status, and complex care needs [2]. Falls, pulmonary aspiration, and dehydration are a particular problem in nursing homes and a major contribution to the deterioration of independence and quality of life of residents [2–7].

The incidence of falls and fall-related injuries, which is a major external cause of death in those living in healthcare institutions, has been reported in numerous epidemiologic studies [8–10]. Choking and aspiration pneumonia are also frequent; choking is the most common external cause of death in residents under 65 years old, while aspiration pneumonia is particularly difficult to diagnose since the moment of aspiration is usually not observed [8–11]. Dehydration, due to poor fluid intake or pathologic loss of body fluids, is considered to be present in 0.8 to 1.4% of nursing home residents. It is associated with frailty, poor cognition, falls, delirium, disability, and mortality and is a major cause of decreased attention and fluctuating mental status, the hallmarks of delirium, in the nursing home [12].

To provide high-quality care and prevent these risks, nursing home multidisciplinary team members (i.e., physicians, nurses, professionals from rehabilitation team, psychologists, social workers, and medical assistants) need to work as a coordinated team, have an effective system of communication with access to information, and understand the residents’ needs [13, 14].

The use of risk prompt displays by nursing home patients may improve the access to information and communication between healthcare professionals, which may in turn reduce the number of falls, dehydration, and pulmonary aspiration events. As soon as a patient is admitted, the nursing team would be responsible for screening for falls, dehydration and pulmonary aspiration risks, and for suggesting the corresponding risk prompt displays that should be used. The local multidisciplinary team would validate the nursing team’s suggestions before a patient would start using the risk displays in their daily routine in the nursing home.

The aim of this study is to develop and evaluate the feasibility of a set of risk prompt displays to communicate the risk of falls, dehydration, and pulmonary aspiration and to reflect on tailored interventions that would appropriately manage these events in nursing homes. In order to do this, a national, single-center, feasibility study will be conducted. The duration of the study for each participant will be a minimum of 3 months.

## Methods

### Study setting

Participants will be recruited from Campus Neurológico Sénior (CNS), a neurological nursing home, located in Torres Vedras, Portugal, which has an outpatient clinic and a residential unit for short or long-term admissions of neurologic patients or individuals aged over 65 years. CNS places its focus on comprehensive care to patients, implemented by a multidisciplinary healthcare team that includes physicians, nurses, a rehabilitation team, dieticians, psychologists, and medical assistants.

### Eligibility criteria

All patients from the CNS residential unit (*n* = 78) who fulfil the following inclusion criteria will be invited to participate:

- Men or women residing at the CNS nursing home for long-term care;
- Risk of falling and/or dysphagia and/or dehydration defined by brief screening assessment;
- Willing to participate in the study;
- Willing to provide written informed consent;
- Willing to comply with the monthly required interviews.

Exclusion criteria include the presence of significant active psychiatric problems (e.g., hallucinations, confusion, psychosis) that, according to the clinical judgement of the CNS multidisciplinary team, could be aggravated with the use of risk reminders.

### Recruitment and intervention

#### Study materials (Figs. 1 and 2)

Created by the CNS risk prompt display study group, the risk reminders are intended to be used by residents, from the moment of admission, in daily routines at the CNS (following validation with the present feasibility study). They include the following standardized materials:

- Small, lightweight rubber coloured bracelets, for patients’ use (on the wrist, visible), with phrases related to the different risks: “prevent rather than fall”, “contain to protect”, “drink to hydrate”, and “avoid choking”.
- Small and coloured signposts next to the patients’ headboard.

**Fig. 1:**
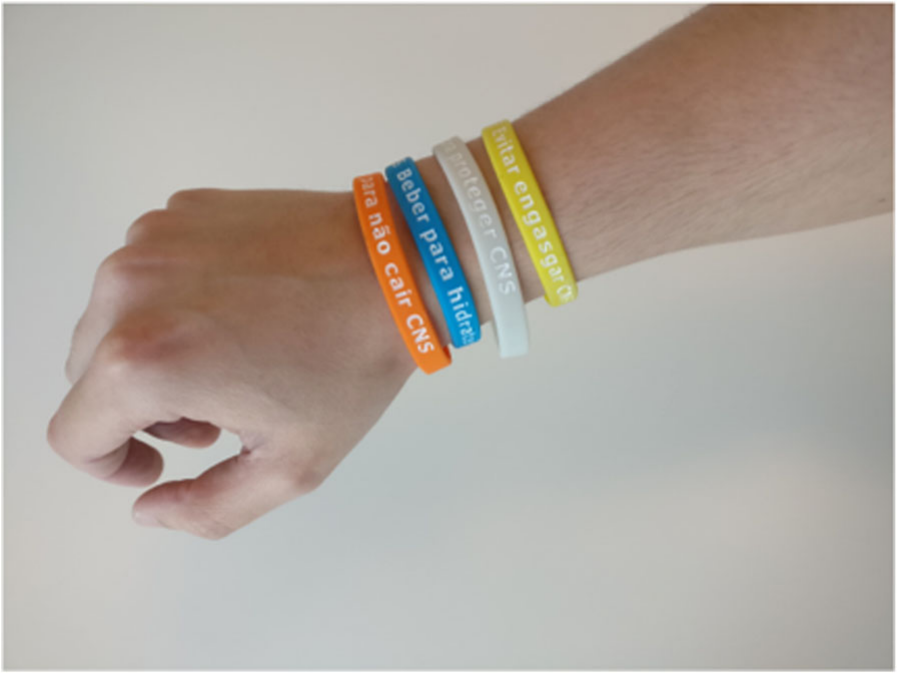
Risk prompt displays—coloured bracelets with slogans related with the different risks

**Fig. 2:**
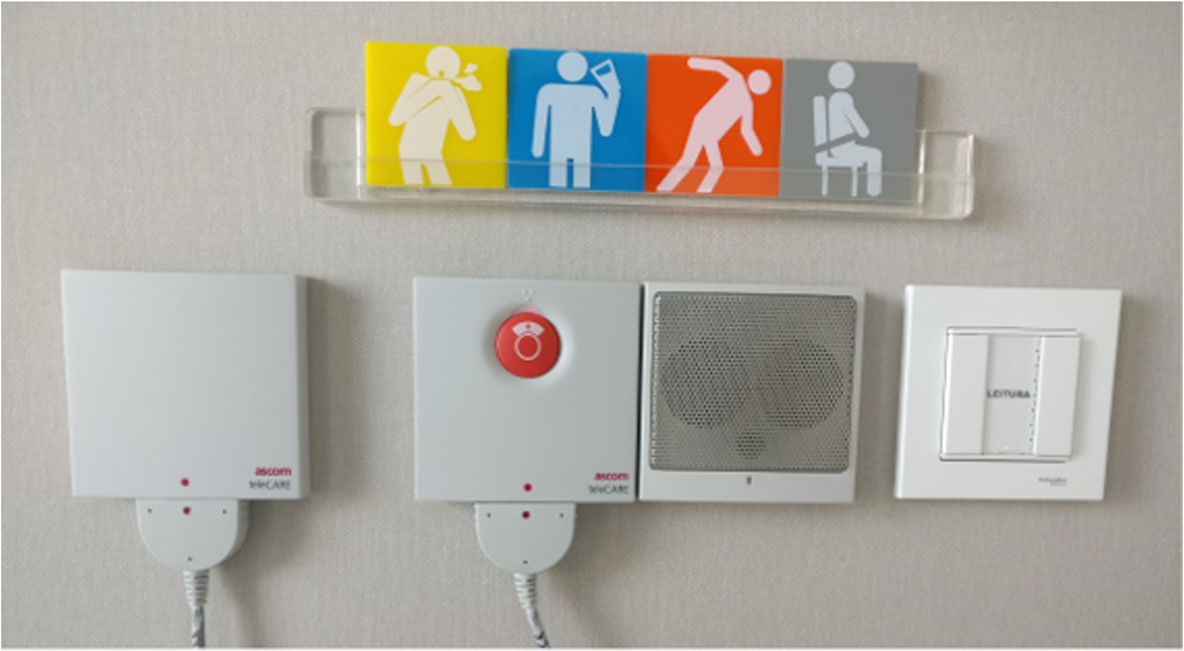
Risk prompt displays—coloured signposts next to patients’ headboard

### Procedures

#### Preparation phase—teaching session for healthcare professionals

One member of the research team will hold an initial multidisciplinary teaching session to train and familiarize the CNS multidisciplinary team with the use of sign displays and the corresponding intervention procedures. Training is expected to last 60 min and to be both a theoretical and practical session. It will include explanations of what constitutes a fall, pulmonary aspiration, and dehydration, and will also include specific instructions on which procedures to undertake at each situation of risk. Additionally, the researcher will provide a practical demonstration of the procedure and any queries from the team will be answered.

#### Screening

All patients will be invited to participate if they fulfil the aforementioned inclusion criteria. The nursing team from the CNS will propose patients for recruitment. Patient risk assessment and inclusion in the study will require clinical assessment and a history taking, before validation by the CNS multidisciplinary team.

For the purpose of participant recruitment, a fall is defined as a sudden, unexpected event that results in coming to rest unintentionally on the ground or at some other lower level [15, 16]. A near fall is defined as an involuntary or uncontrolled descent not ending on the ground or at some other lower level [17]. Given our interest in the phenomena and not the cause, all falls, either resulting from environmental hazards or overwhelming external force, disease-related symptoms, and/or attributable to acute medical events such as syncope and seizures, will be considered.

A dysphagia or pulmonary aspiration event will be defined as inefficient or unsafe transfer of food, liquid, or saliva from the mouth into the stomach [18].

Dehydration will be defined by the loss of body water, with or without salt, at a rate greater than the body can replace it [19].

At the end of screening and risk assessment, the corresponding risk messages will be given to the patient by the investigator.

If patients are unable to give informed consent, legal guardians will be asked for consent and authorization of a screening visit. Informed consent containing comprehensive information about objectives, duration, procedures, willingness, and possible risks to study participation, will be obtained from patients before any study-related proceedings. Patients will be encouraged to take time to think and clarify any doubts they may have before signing informed consent and, when they feel ready, to communicate the decision to one of the members of the research team. During the screening visit, an explanation of the objectives and compliance needed for the study will be given to the participants and caregivers and all questions will be considered and answered.

#### Baseline assessment

Demographic data, clinical manifestations and disease management, comorbidities, and past medical conditions will be obtained using a structured questionnaire. In addition, a brief clinical assessment of risk of falls, pulmonary aspiration, and dehydration, including the clinical scales listed below, will be performed.

### Mini Mental State Examination (MMSE) [20]

The MMSE is a brief 30-item questionnaire that is used to quantitatively assess cognition. The MMSE test consists of 11 simple questions grouped into 7 cognitive domains: the time and place of the test, repetition of three words, attention and calculation, recall of three words, language use, and visual construction. It can be used to screen for cognitive impairment (cut-off scores: none: 24–30; mild: 18–24; and severe: 0–17), to estimate the severity of cognitive impairment at a given point in time, to follow the course of cognitive changes over time, and to document an individual’s response to treatment.

### Timed Up and Go Test (TUG) [21–23]

The TUG is a quick capacity measure to assess frailty, functional mobility and is a good predictor of an individual’s ability to independently walk outside safely. It requires that the participant get up from a standard chair, walk 3 m at a comfortable and safe speed and then turn walk to back to sit in the chair. The TUG is recommended in the latest physiotherapy guidelines and by the International Parkinson and Movement Disorder Society (MDS) Rating Scales Committee as an instrument to assess posture, gait, and balance in Parkinson’s disease. It is also recommended as a tool to identify frailty and risk of fall in the older population (cut-off score indicating risk of falls > 13.5 s).

### Morse Fall Scale [24]

The Morse Fall Scale assesses the risk of falling for hospital inpatients or those in long-term care. In particular, it evaluates fall history, the presence of comorbidities, the use of walking aids, mental status, and whether or not patients are receiving intravenous therapy. Each criterion evaluated receives a score ranging from zero to 30 points, summed to provide a risk score, which is classified as follows: low risk, from 0 to 24; mean risk, 25–44; and high risk, ≥ 45.

### Swallowing Disturbance Questionnaire (SDQ) [25, 26]

The SDQ for Parkinson’s disease patients is a validated self-reporting 15-item questionnaire on swallowing disturbances that appear in the oral and pharyngeal phases of swallowing. Fourteen questions rated on a four-point scale (0 for no disturbance and 3 for severe disturbance) and one dichotomous question (“yes/no”; yes is scored 2.5 and no is scored 0.5). If the patient is unable to respond, a CNS nurse familiar with the patient’s history will complete this questionnaire.

### GULP dehydration risk screening tool [27]

The GULP is a screening tool to assess geriatric dehydration risk. It includes a score from 0 to 7 points for three categories (24 h fluid intake; urine colour; clinical risk factors for dehydration). Based on results, the GULP tool recommends a hydration management plan, thereby engaging the patient in self-monitoring of urine and verbal prompts.

#### Diary records

At the end of each nursing shift all events—falls, near falls, dehydration, and pulmonary aspiration—will be recorded in documents specifically created for this purpose, by the CNS risk prompt display study group.

#### Monthly visits

At the end of each month, patients will undergo an interview performed by a nurse (member of the research team), and a self-completed questionnaire will be handed to healthcare professionals. Both will be questioned regarding overall acceptability levels and asked about any problems encountered regarding the use of the risk prompts. Additionally, these moments will include questions on clarity of the instructions, a reminder of the event definitions and the registration procedures.

In order to avoid excluding demented patients, if the patient is unable to answer the questions, only the occurrence of adverse events and the reason for not performing the interview will be recorded.

#### Monthly reports

At the end of each month, as a strategy to keep healthcare professionals informed and to ensure they remember the importance of registering events, a newsletter will be sent to all CNS staff. This will contain information about the study duration and will present a summary of the number of participants in the study (number of patients recruited and number of drop-outs) and of the falls, dehydration, and pulmonary aspiration events recorded in that month.

#### Data collection of events

For all CNS residents, we will also collect historical data related to falls, dehydration, and pulmonary aspiration events retrospectively from medical and nursing charts within the period of the study and for the same period the year before.

### Outcomes

#### Primary outcome

To evaluate the feasibility of risk prompt displays designed to communicate risks.

#### Secondary outcomes

The secondary outcomes will be to evaluate the following:

- Patients’ and health professionals’ satisfaction;
- The efficacy of risk prompt displays in reducing the number of falls, pulmonary aspiration, and dehydration events;
- The type and frequency of adverse events that arise from using the risk prompt displays (for example, physical or social discomfort, skin irritability, stress, or anxiety).

### Statistical analysis

Statistical analysis will be performed using SPSS® version 21.0; SPSS Inc. Chicago, IL [28]. Data will be described using descriptive statistics, i.e., means, medians and relative frequencies (i.e., outcome per number of days of hospitalization).

As this is an exploratory study with no previous studies having been conducted, we defined 20 patients as enough to conclude on the primary outcome on feasibility. This is a compromise between the capability to respond to a specific question and a realistic perspective of recruitment capacity using just one nursing home [29].

As our primary outcome, we will measure patients’ adherence to the risk prompts through the number of times and reasons they remove the risk prompts or refuse to use prompts during the period of the study and/or withdrawing from the study.

The secondary outcomes will be the following:

1. Patients’ satisfaction with the risk prompt displays measured on a 7-point Likert scale and open-ended questions.
2. Healthcare professionals’ satisfaction with the risk prompt displays as measured on a 7-point Likert scale and open-ended questions regarding the overall benefits of using the displays and overall perception of its impact on decreasing risk.
3. Historical comparison of medical and nursing charts data on the relative frequency of number of events per number of days of hospitalization registered during the period of the study, with the same period the year before.
4. Type and frequency of adverse events recorded during the period of the study.

### Data record

Study documents will be archived at the CNS in a way to ensure (1) their integrity once placed within the archive, (2) to prevent unauthorized access, and (3) to guarantee that they are readily available, upon request, by the competent authorities. The information will be confidentially handled in order to prevent subject names or other directly identifiable information appearing in any reports, publications, or other disclosures of clinical study outcomes. The investigator will assign a code to each participant and save personal information (name, birth, contacts) in a different database from the one in which statistical analysis will be performed.

## Discussion

Thoughtful selection of proper risk prompt displays in nursing homes is an essential step along a path toward efficient communication of risks among teams. We expect that the displays will potentially improve communication given their simplicity, discretion, low complexity, and minimal physical requirements.

The results of the study will enable the planning, at a later stage, of clinical studies designed to respond to more pragmatic questions regarding the use of risk prompt displays, such as (1) efficacy of the use of the risk prompt displays, (2) better clinical trial design to evaluate the efficacy of displays, including outcomes, (3) a better treatment strategy including the combination of non-pharmacological therapies with the use of the risk prompt displays, and 4) introduction of the use of a risk prompt bracelet in the therapeutic areas studied.

A limitation of this study is that the teaching session or the monthly reports encourage event registration, resulting in an apparent increase of events during the period of the study. For that reason, we will widen data collection to all CNS residents, and will also collect data of events in the same period, 1 year before.

This study will only be conducted in one nursing home, which could be a limitation. However, the CNS population is representative in age, heterogeneity of diagnosis, and symptoms of other nursing homes. As this is a protocol for a first exploratory study, we anticipate that running the study in just one nursing home will not compromise the capability to conclude on patients’ adherence.
